# Persistence in the Methadone Maintenance Program and Its Relationship with the Medication Regimen Complexity Index in Opioid-Dependent Patients

**DOI:** 10.3390/ph17050567

**Published:** 2024-04-29

**Authors:** Elena Alba Álvaro-Alonso, María del Carmen Gómez-Álvarez, Beatriz Segovia-Tapiador, María Isabel Del-Pino-Illaconza, Jorge Valencia, Pablo Ryan, Antonio Aguilar-Ros, Ismael Escobar-Rodríguez

**Affiliations:** 1 Pharmacy Department, Infanta Leonor University Hospital, Av. Gran Vía del Este, 80, 28031 Madrid, Spain; ismael.escobar@salud.madrid.org; 2 General Subdirectorate of Pharmaceutical Inspection and Management, Authorization Area for Pharmaceutical Centers, Services and Establishments, Ministry of Health, C/Aduana, 29, 28013 Madrid, Spain; mariacarmen.gomez.alvarez@salud.madrid.org; 3 Vallecas Comprehensive Care Center for Drug Addicts, Calle de las Cinco Villas, 5, 28051 Madrid, Spain; beatriz.segoviat@salud.madrid.org (B.S.-T.); isabel.delpino@salud.madrid.org (M.I.D.-P.-I.); 4 Internal Medicine Department, Infanta Leonor University Hospital, Av. Gran Vía del Este, 80, 28031 Madrid, Spain; jorge.valencia@salud.madrid.org (J.V.); pablo.ryan@salud.madrid.org (P.R.); 5 Instituto Universitario de Estudios de las Adicciones IEA-CEU, Universidad San Pablo-CEU, CEU Universities, Urbanización Montepríncipe, 28660 Boadilla del Monte, Spain; aguiros@ceu.es

**Keywords:** methadone maintenance program, medication regimen complexity index, adherence, persistence, opioid-dependent patients

## Abstract

It has been shown that the Medication Regimen Complexity Index (MRCI) is a useful and reliable tool for calculating the complexity of the pharmacotherapeutic regimen (CPR). Furthermore, a high MRCI is associated with lower adherence. However, the MRCI of opioid-dependent patients (ODP) has not been studied. The aim of this study is to calculate the Methadone Maintenance Program (MMP) persistence and the MRCI score in a ODP cohort. Second, to analyze its relationship and association with other variables. To accomplish this research, an observational study including adults with a confirmed diagnosis of opiate-dependency according to the DSM-5 in a MMP center was carried out. To define MMP-persistence, a group was created by the researchers who defined five weighted items according to their agreed importance. Our first contribution was to create a new definition of MMP-persistence. This study also identified age, comorbidities, and received methadone maintenance doses as successful predictors for MMP-persistence. We have also shown that the MRCI does not seem to be a useful tool to determine MMP-persistence, probably because there are multiple factors that influence it in addition to the CPR. It is necessary to continue searching for more precise selection and stratification tools for ODP to improve their persistence.

## 1. Introduction

In the Autonomous Community of Madrid, Resolution 189/2018 [1] was published in February 2018, entrusting the Pharmacy Service of the Infanta Leonor University Hospital (SF-HUIL) with the task of daily supply of methadone to the 27 Centers for the Comprehensive Care of Drug Addiction Patients (CAID) within the Madrid Health Service. The aim of this resolution was to centralize the acquisition, preparation, distribution, and dispensing of methadone by the SF-HUIL. This initiative represented the first step in changing the pharmacotherapeutic health care model for the treatment of the patients in the program. The SF-HUIL began this activity in March 2018. This was the first time that hospital pharmacists were responsible for this type of function in the field of addiction treatment, which was a pioneering experience in the Autonomous Community of Madrid.

After consolidating the first and second phases of this project [2,3,4] (acquisition management, methadone elaboration, distribution to CAID and galenic development), it was time to take on more clinical and pharmaceutical care actions including information and training aimed at CAID professionals and patients.

Several factors can determine adherence to pharmacotherapy, such as the patient’s socioeconomic characteristics, loss of cognitive ability, prescription of complex regimens, and the occurrence of adverse effects. Among these factors, the complexity of the medication regimen has been identified as one of the main determinants of non-adherence as it directly affects the patient’s ability to follow treatment instructions. In fact, it is known that more complex medication regimens are associated with lower medication adherence, especially among the elderly, adults, and patients with chronic non-communicable diseases [5,6,7,8,9,10,11], therefore being associated with a greater risk of therapeutic failure, hospital readmission, and mortality.

Currently, the most widely used validated instrument to assess the complexity of medication regimens is the Medication Regimen Complexity Index (MRCI) [12], which was developed by George et al. [13] from a cohort of patients with chronic obstructive pulmonary disease (COPD). It is a 65-item tool that assesses and quantifies the characteristics that make a pharmacotherapeutic regimen complex, beyond just taking into account the number of medications a patient takes, since, although this contributes to complexity, it does not constitute complexity per se [14,15]. The MRCI was adapted and validated into Spanish to obtain the first version of the pharmacological treatment complexity index adapted to Spanish (MRCI-E) [16]. This validation was also carried out by the research team of the SF-HUIL.

The use of the MRCI allows us to quantitatively measure the complexity of the regimen to define strategies in selected patients [17,18] and carry out simplification, education, supervision, or support interventions that make it easier for patients and their caregivers to comply with their specifications to improve adherence and health results [19,20,21,22,23].

To sum up, although it has been shown that the MRCI is a useful and reliable tool for calculating the complexity of the pharmacotherapeutic regimen of different population groups with chronic illnesses such as HIV infection, diabetes, depression or COPD, until now, its use in drug-dependent patients undergoing methadone treatment has not been evaluated or published, so this would be the first study in the literature with this objective. Furthermore, MRCI can help identify patients who are at increased risk of adverse events and could be used to support clinical decision making to improve medication management and the health outcomes of the patients.

This fact is what led us to design our study; its purpose was to calculate the MRCI in opioid-dependent patients undergoing methadone treatment and to analyze its relationship with adherence to the Methadone Maintenance Program (MMP).

## 2. Results

Regarding the bibliographic search, there are numerous and varied studies [24,25,26,27,28,29,30,31,32,33,34,35,36] in the literature from different geographical areas, mainly Asian, Canadian and American, from different healthcare settings, mainly involving outpatients in detoxification clinics and community pharmacies, that investigate different issues related to adherence to methadone maintenance treatment (MMT). However, the main limitation of these studies is that they interchangeably express adherence or retention to MMT or MMP, the latter including interventions beyond the mere collection of daily methadone doses.

The focus group that designed and created the definition of MMP adherence redefined as MMP persistence. This new concept established that a patient was MMP persistent if they obtained a score of ≥90%, considering five items weighted according to the importance agreed upon in the focus group (Table 1).

Regarding the observational study, of around 200 patients who were offered participation, a total of 84 patients finally signed the informed consent, so they were included. Table 2 presents the population characteristics for the patient sample.

The majority of the patients were in their 50s, and the cohort was mainly composed (79.8%) of men.

A total of 57% had any comorbidity that required a doctor follow-up, including pathologies such as COPD, high blood pressure, diabetes mellitus, cardiovascular disease, etc.

Regarding the addiction profile, we can affirm that this is a cohort of polydrug addict patients.

In relation to methadone treatment, 52.38% of the patients received a daily dose ≥ 60 mg (63.1% in maintenance dose). In relation to pharmacotherapeutic complexity, 66.7% patients received polypharmacy, accounting for a MRCI total score of 13.5 (maximum score of 40.5). Section B (frequency) has the most weight of the total MRCI score.

Regarding persistence to the MMP, 77.4% of patients were persistent in the study, according to our definition.

Once the study cohort was described and characterized, we proceeded to study the relationship between the MRCI score and persistence to MMP, finding no association (*p* = 0.74).

Next, we studied the two subpopulations (persistent and non-persistent to MMP) and the association of persistence to MMP with the different study variables (Table 3).

The age of persistent patients is, in general, older than that of those who are not persistent: 49.23% vs. 42.1% between 50 and 60, and the 12 patients from the general population who were over 60 years old are in this group of persistent patients. Regarding the dose, within the subgroup of persistent patients, we found that 56.92% received a dose ≥ 60 mg daily, while in the subgroup of those not persistent to MMP, only 36.84 % received a similar dose.

As can be seen, a statistically significant relationship has been found between persistence to MMP and the following variables: age (*p* = 0.04), comorbidity (*p* = 0.002), and receiving methadone in maintenance doses (*p* = 0.024). And, although it was nearly statistically significant (*p* = 0.053), active heroin use was also related, although negatively, to persistence to MMP.

The results obtained in the multivariate analysis are shown in Table 4.

It is worth highlighting the heroin consumption (OR: 0.185; *p* = 0.028), which was significantly associated with less MMP persistence. The model showed a good fit (Hosmer–Lemeshow test *p* = 0.244).

Regarding the medication complexity (defined as MRCI ≥ 15, taking into account the median obtained in the patients of our study), the mental health disorders (OR: 15.928; *p* = 0.008) and comorbidities (OR: 7.200; *p* = 0.052) obtained a statistically significant association in the multivariate analysis. However, heroin use (OR: 0.231; *p* = 0.10), although it was not statistically significant, has been shown to be a protective factor for the complexity of the pharmacotherapeutic regimen. The model showed a good fit (Hosmer–Lemeshow test *p* = 0.567).

## 3. Discussion

This study has allowed us to describe and characterize our opioid-dependent patients undergoing methadone treatment cohort, to define the concept of MMP adherence (which is not clearly defined in the literature) and to verify whether the MRCI is a valid tool for the intervention of the specialist pharmacist to improve patients’ MMP adherence through different strategies.

MMP retention has been found to be correlated with adherence [37] and both are known to improve an individual’s health [38,39] as they lead to abstinence from opioid use [40]. However, retention is different from adherence. Be that as it may, inconsistent adherence to MMP can be problematic, as this can increase susceptibility to overdose [41]. The first contribution of our study was the design and creation of a new and unique definition of adherence to the MMP, redefined under the name “persistence to MMP”.

This new definition has other advantages, such as the weighting of the items that compose it. The group of experts considered that the first three items: A, B and C, were the determinants that had to be inexcusably met to consider a patient persistent to the MMP. For this reason, and when considering persistence as a total score ≥ 90%, all of them had to have a value greater than 10%. This meant that items D or item E were the only items the patient did not necessarily have to comply with. Both, therefore, scored 10%. And the fact is that, for the group of experts, these two factors, although no less important than the previous ones, were less decisive in considering a drug-dependent patient as persistent to the MMP. Furthermore, a period of time of three months was taken into account, and not the last month or any scale, as occurs in numerous studies in the literature [27,28,31,32,33,34,35]. This allows us to reflect the patients’ activity in a more continuous period of time, rather than a specific moment that could not really define their situation, and thus lead to concluding that the patient is not persistent to the MMP due to some exceptional issue. In this way, the calculation of this new definition allows us to obtain a result that is much more adjusted to the reality of persistence to the MMP, taking into account its global nature. Therefore, this new and more complete definition could serve as a reference in the field of studying the adherence of drug-dependent patients. On the other hand, it should also be noted that, although the new definition of MMP persistence was created by the consensus of a focus group of experts, it would be desirable for this definition to be validated.

On the other hand, in our study, a high percentage of patients persistent to the MMP was obtained (77.4%). This finding was much higher than that found in other studies carried out in large cities in Asian countries such as Vietnam (42.1%) [31,33,42] or China (from 11.8 to 25.8%) [43,44] or in European cities and countries, such as London (42%) and France (46%) [28]. However, it is similar to other works carried out in Indonesia (74.2% and 61.3%) [45] and some cities in the United States such as Denver (60%) [46] and Montreal in Canada (78%) [47]. The main difference lies in whether the studies have carried out the analysis of methadone adherence or whether MMP retention has been calculated. The latter are those that share similar results to those found in our work. Therefore, the difference between both concepts is notable, as previously mentioned.

We want to highlight the high proportion of smokers and alcohol consumers among the patients in our study, whose figures are similar to other studies [31]. Although this association did not obtain statistical significance in our results, it has been shown that patients receiving methadone treatment could suffer from diseases related to smoking and alcohol consumption [48,49] as well as lower adherence to methadone treatment [28,50,51]. This result suggests that personalized counseling to quit smoking and stop drinking alcohol should be applied and/or enhanced in CAID [28,52]. The same can be applied to mental health disorders. It is a very important variable to take into account to obtain more success with methadone treatment, and to increase compliance and ensure retention in the MMP [33,35]. For example, depression has a negative impact on treatment outcomes in patients treated with methadone [53].

Continuing with the analysis, we can infer that the patients most likely to be persistent to the MMP, are those of older age, who receive methadone in maintenance doses and who have some comorbidity that requires medical follow-up, these being, therefore, variables or determinants of success for MMP persistence. It is also notable that heroin consumption, although with a *p* value close to statistical significance, has been negatively related to persistence to the MMP, being, therefore, a determinant of failure.

These associations make sense, as they are patients who, on the one hand, receive methadone in maintenance doses, which implies that they are stable and can control their addictive situation. On the other hand, by having comorbidities that require medical monitoring, patients are more closely monitored by health professionals who contribute to patient retention in the MMP. And finally, older age, in general, implies greater experience on the part of the patient, which can impact better self-care and therefore greater persistence to MMP. This is understandable because, compared to younger patients, older people are more mature and tend to be more likely to be responsible for themselves and their families.

These results are consistent with other previously published results confirming that older patients had a better response and retention in MMP, while younger patients are more likely to abandon the substitution treatment for opioid dependence [28,29,31,45,54,55,56]. We also want to highlight that, just as in some systematic reviews and controlled trials, in which it was shown that the daily dose of methadone was the main factor in adherence to MMP [28,29,45,46,54,57,58,59,60], in our study we have also demonstrated this relationship by finding that patients who received methadone in maintenance doses were more persistent to MMP. Furthermore, in the literature it has been found that lower adherence to the MMP is associated with methadone doses lower than 60 mg/day [29,57,58], as occurred in our study.

With respect to the consumption of other illicit drugs, in our study, heroin consumption has been negatively related to persistence to MMP, this result being consistent with the literature [31,61]. However, studies have also been found in which it has been shown that heroin use is positively associated with adherence to methadone treatment [27,28]. This is the case in the recent study by Wang et al. [27], in which a higher level of craving for heroin was associated with a higher level of adherence to methadone treatment. This is because heroin users were voluntarily motivated to undergo methadone treatment, in addition to the fact that methadone helps alleviate this desire and, therefore, reduce heroin consumption. This suggests that some negative behavioral characteristics before initiating methadone maintenance treatment do not predict negative outcomes and should therefore not be a barrier to MMP initiation.

Other MMP retention predictors found in the literature include type of employment, income, education level, marital status, having been in prison, ethnic race, and even living in rural areas [31,54,55,56,62]. However, these variables have not been included in our study because they were not decisive for our objective.

The second part of our study consisted of analyzing the correlation of pharmacotherapeutic complexity using the MRCI index and persistence to the MMP. Contrary to expectations [5,6,7,8,9,10,11], no statistically significant association was found. This means that a high MRCI score, by itself, is not sufficient to be related to MMP persistence and that, therefore, the MRCI does not seem to be a useful tool to determine persistence to MMP, probably because there are multiple factors (social, family, methadone dose, age, etc.) that influence this persistence beyond the complexity of pharmacotherapy, as described above. It could also be due to the small sample size of our study.

We have also found that, in general, two-thirds of the patients studied receive polypharmacy. However, the lack of correlation between MRCI and persistence should not be an obstacle to implementing measures to optimize pharmacotherapeutic complexity in these patients.

Continuing with the analysis, the drug-dependent patients that are more likely to have a higher MRCI index score and, therefore, have greater pharmacotherapeutic complexity, are those who are older, homeless, with some comorbidity that requires medical monitoring, with HBV infection and/or a mental health disorder, who actively consume heroin and have used the parenteral route to consume in the last year. And of course, all those patients who are in a polypharmacy situation and are receiving more than five medications.

Therefore, we have identified the group of patients who would first benefit from different interventions by CAID professionals and in whom the hospital pharmacist is key to reducing their pharmacotherapeutic complexity.

Regarding strategies to reduce MRCI, we have found some studies [14,19,21] which offer an approach to simplifying medication regimens through the figure of a pharmacist who reviews the treatment and makes recommendations to the prescribing physician [20,21,63]. It is also interesting to note that, for greater success, it would be necessary to involve the primary care physicians who see these patients, to ensure that the changes proposed by the pharmacist or the CAID doctor have continuity of care [22].

Nevertheless, despite the fact that this study can serve as a model for a starting point in pharmaceutical care for this type of patient within the Community of Madrid, it has certain limitations. This is a single-center study with a small sample size, which limits the generalizability of the findings. A larger, multi-center study would provide more robust results that could be applied more broadly to opioid-dependent patients on methadone maintenance treatment. The data for calculating the MRCI score were collected retrospectively from the electronic medical record without information provided by the patient; therefore, it is not possible to verify if the list of treatments is up to date, or if the patients were taking some medications for other purposes or self-medicating, so important information may be missing or inaccurately recorded. Prospectively collecting data directly from patients could improve data quality. A common limitation with other published studies is that they only include data on officially prescribed medications and do not include alternative treatments or medicines from the private healthcare system. However, we do not consider it a very significant limitation in our study, given the universal coverage of the public health system in Spain, with a small number of patients using alternative medications. Additionally, the electronic medical record data does not capture instructions that a patient may have received verbally from medical professionals or written material provided to the patient (e.g., take medication on an empty stomach), thus providing a lower MRCI score. Finally, the study used a single MRCI evaluator based on a retrospective chart review, so it was not possible to assess inter-evaluator agreement of the MRCI tool. Having multiple evaluators independently calculate MRCI and assess inter-rater reliability would strengthen the MRCI results. Patient interviews could also help capture additional information impacting medication complexity. Finally, the cross-sectional study design prevents determination of causality between the factors analyzed and persistence in the methadone maintenance program. A longitudinal cohort study following patients over time could better elucidate predictors of treatment persistence.

In the future, one of the lines of research would be to look for other alternative tools to the MRCI index that can be correlated with the persistence to MMP of drug-dependent patients, or even the use of machine learning techniques to optimize and improve accuracy, which could inspire novel approaches to predicting persistence in methadone maintenance treatment, beyond the medication regimen complexity index.

## 4. Material and Methods

### 4.1. Design, Setting and Participants

A descriptive observational study was designed that included adult patients with a confirmed diagnosis of opiate dependence according to the DSM-5, undergoing methadone treatment at the CAID Vallecas in the Community of Madrid. The study was carried out from November-2021 to April-2022. There were no exclusion criteria.

### 4.2. Variables

Descriptive variables such as sex, age, socio-labor situation, comorbidities, active drug consumption, different aspects of methadone treatment (doses, frequency, duration), as well as the number of dropouts and/or losses to follow-up since the MMP onset, were collected.

For this, medical records of the electronic medical history program Horus^®^ and Selene^®^ were used. To find out the patient’s pharmacotherapeutic history, the Single Prescription Module (MUP^®^) was consulted and, to know the treatments for hospital use (antiretrovirals… etc.), the SF-HUIL programs were consulted.

The MRCI score was also calculated using as a model the MRCI-E calculator validated in Spanish [16].

The study data were collected and managed using REDCap^®^ [64,65].

### 4.3. Adherence to Maintenance Methadone Program

A bibliographic search was performed in order to define the concept of MMP adherence, consulting different databases: Medline, Embase, Cochrane Library, Wiley Online Library and Pubmed. In this search, several Medical Subject Headings (MeSH) were used: “medication adherence”, “retention in care”, “methadone”. In addition, the search was completed with the use of keywords such as “predictors”, “factors” “opioid dependence” or “methadone maintenance”.

Then, both its definition and the variables with which it was related were analyzed.

As will be explained later in the results section, no definition of MMP adherence was found in the literature beyond the classic concept of methadone adherence, understood as collection of methadone at a specific time. To this end, it was decided to create a multidisciplinary focus group with the collaborating researchers of the study (doctors, pharmacists, clinical psychologists, and nurses from CAID Vallecas and Infanta Leonor University Hospital) in which, by consensus, adherence to the MMP was defined.

### 4.4. Statistical Analyses

The qualitative variables were presented with their frequency distribution and percentage, while the quantitative variables were presented with their mean and standard deviation, when they follow a normal distribution and with median and interquartile range in non-normal situations.

The relationship between the continuous variables and the dichotomous qualitative variables was determined using Student’s T test for independent samples, after Levene’s homogeneity of variances test, when the variables follow a normal distribution in the groups to be compared, and the non-parametric test of the Mann–Whitney U was used otherwise. In the case of more than two groups, ANOVA or its corresponding non-parametric Kruskal–Wallis test was used.

The association between the different variables with the MMP persistence and the MRCI score was carried out by constructing a logistic regression model with the Pearson correlation coefficient or the Spearman non-parametric coefficient, which was most appropriate for each situation. In addition, variables that were associated with a *p* < 0.1 in the univariate analysis were included in the multivariate analysis. The goodness of fit of the logistic regression model was checked with the Hosmer–Lemeshow test.

The minimum number of patients necessary to obtain statistically significant association was not calculated since the study was offered to all patients at the center.

For the statistical analysis, the IBM SPSS Statistics^®^ Version 27 program was used and an alpha value ≤ 0.05 was considered significant for all analyses.

### 4.5. Ethical Considerations

This study has been carried out respecting the principles and basic ethical standards that have their origin in the current revision (revised version of Fortaleza, 2013) [66] of the Declaration of Helsinki, approved by the World Medical Assembly and with the current regulatory requirements included in Spanish Royal Decree 957/2020, of November 3, which regulates observational studies with medicines for human use [67].

This study had the favorable opinion of the Ethics Committee for Research with Medicines (CEIm) of the Gregorio Marañón University General Hospital and informed consent was obtained from all patients prior to their inclusion in the study.

## 5. Conclusions

A new and unique definition of persistence has been designed and created for the MMP. Age, having any comorbidity that requires medical follow-up, and receiving methadone in maintenance doses, have been identified as successful predictors of persistence to MMP. The MRCI may not be a viable tool for determining MMP persistence. This finding should serve to continue the search for more precise tools for the selection and stratification of patients included in the MMP to improve their persistence.

## Figures and Tables

**Table 1:** Definition of the new concept of persistence to the MMP. Description and weighting of each item.

Item Description	Weighing (%)
A. Collection of daily methadone doses by more than 90% in the last 3 months.	40
B. Negative urine toxicological analysis for opioids in more than 90% in the last 3 months or that no sample has been collected for toxicological control in the analyzed period.	20
C. Attend more than 90% of the appointments scheduled with the doctor in the last 3 months or have not had any appointments in the aforementioned period of time.	20
D. Attend more than 90% of the appointments scheduled with the clinical psychologist in the last 3 months or have not had any appointments in the aforementioned period of time.	10
E. Negative toxicological analysis in urine for other non-opioid substances in more than 90% in the last 3 months or that no sample has been collected for toxicological control in the period analyzed.	10
Persistence to MMP = A + B + C + D + E =	100%

**Table 2:** Results of the frequencies of the variables collected during the study.

	Global (N = 84)
Man ⴕ	67/84 (79.8)
Age *	51 (46–56) (N = 84)
**Socio-labor situation**	
Job ⴕ	15/59 (25.4)
Without own home ⴕ	19/67 (28.3)
Homeless ⴕ	10/67 (14.9)
Socio-family support ⴕ	56/64 (87.5)
**Comorbidities**	
Comorbidity that required a doctor follow-up ⴕ	45/79 (57.0)
Infectious disease ⴕ	50/80 (62.5)
HIV ⴕ	18/50 (36.0)
HBV ⴕ	10/50 (20.0)
HCV ⴕ	48/50 (96.0)
Active HCV ⴕ	0/48 (0.0)
Mental health disorder ⴕ	29/73 (39.7)
**Addictions**	
Intravenous drug users (IVDU) in the last year ⴕ	2/68 (2.9)
Patient’s situation at the time of signing the IC:	
Stable/Maintenance ⴕ	53/84 (63.1)
Induction ⴕ	2/84 (2.4)
Relapse ⴕ	6/84 (7.1)
Dose shift/decrease ⴕ	9/84 (10.7)
Unknown ⴕ	14/84 (16.7)
Active consumption of toxic substances:	
Alcohol ⴕ	25/59 (42.4)
Tobacco ⴕ	57/70 (81.4)
Cannabis ⴕ	17/60 (28.3)
Cocaine ⴕ	39/60 (65.0)
Heroin ⴕ	20/59 (33.9)
Smoked heroin ⴕ	16/17 (94.1)
Intravenous heroin ⴕ	1/19 (5.3)
Benzodiazepines ⴕ	54/73 (74.0)
**Methadone treatment**	
Daily dose of methadone (mg) *	60 (40–80) (N = 84)
Methadone dosage	
Every 24 h ⴕ	84/84 (100.0)
Every 12 h ⴕ	0/84 (0.0)
Every 8 h ⴕ	0/84 (0.0)
Methadone pharmaceutical form	
Oral solution 10 mg/mL ⴕ	79/84 (94.0)
Methadone tablets ⴕ	5/84 (6.0)
Time on methadone treatment	
Less than 1 year ⴕ	10/84 (11.9)
Between 1 and 5 years ⴕ	20/84 (23.8)
Between 5 and 10 years ⴕ	22/84 (26.2)
More than 10 years ⴕ	32/84 (38.1)
Number of MMP dropouts *	0 (0–0) (N = 84)
Number of interruptions/losses to follow-up *	1 (0–2) (N = 84)
**MRCI score**	
Number of medications *	6 (4–9) (N = 78)
Polypharmacy ⴕ	52/78 (66.7)
Section A MRCI *	3 (3–6.25) (N = 78)
Section B MRCI *	8.5 (4.87–12.62) (N = 78)
Section C MRCI *	1 (1–2.25) (N = 78)
TOTAL score MRCI *	13.5 (8.5–21.8) (N = 78)
**Persistence to MMP**	
Total persistence rate to MMP *	100 (90–100) (N = 84)
NON persistent patients ⴕ	19/84 (22.6)

* Continuous variables (median, Interquartile Range, N); ⴕ Categorical variables (n/N, %); IC: Inform consent.

**Table 3:** Results based on persistence to MMP.

	Persistent to MMP (N = 65)	NO Persistent to MMP (N = 19)	*p* Value
Man ⴕ	52/65 (80.0%)	15/19 (78.9%)	0.92
Age *	52 (48–58) (N = 65)	48 (43–52) (N = 19)	**0.04**
**Socio-labor situation**			
Job ⴕ	14/45 (31.1%)	1/14 (7.1%)	0.09
Without own home ⴕ	12/51 (23.5%)	7/16 (43.8%)	0.20
Homeless ⴕ	8/51 (15.7%)	2/16 (12.5%)	1.0
Socio-family support ⴕ	42/48 (87.5%)	14/16 (62.5%)	1.0
**Comorbidities**			
Comorbidity that required a doctor follow-up ⴕ	40/60 (66.7%)	5/19 (26.3%)	**0.002**
Infectious disease ⴕ	37/61 (60.6%)	13/19 (68.4%)	0.54
Mental health disorder ⴕ	22/56 (39.3%)	7/17 (41.2%)	0.89
**Addictions**			
Intravenous drug users (IVDU) in the last year ⴕ	1/53 (1.9%)	1/15 (6.7%)	0.40
Patient’s situation at the time of signing the IC:			
Stable/Maintenance ⴕ	42/55 (76.4%)	11/15 (73.3%)	**0.024**
Induction ⴕ	2/55 (3.6%)	0/15 (0.0%)	
Relapse ⴕ	2/55 (3.6%)	4/15 (26.7%)	
Dose shift/decrease ⴕ	9/55 (16.4%)	0/15 (0.0%)	
Active consumption of toxic substances:			
Alcohol ⴕ	19/45 (42.2%)	6/14 (42.9%)	0.97
Tobacco ⴕ	46/54 (85.2%)	11/15 (73.3%)	0.28
Cannabis ⴕ	13/46 (28.3%)	4/14 (28.6%)	1.0
Cocaine ⴕ	30/49 (61.2%)	9/15 (60.0%)	0.93
Heroin ⴕ	12/45 (26.7%)	8/14 (57.1%)	0.053
Benzodiazepines ⴕ	41/55 (74.5%)	10/15 (66.7%)	0.53
**Methadone treatment**			
Daily dose of methadone (mg) *	67.3 ± 41.3	53.4 ± 24.4	0.07
Number of interruptions/losses to follow-up *	1.03 ± 1.26	1.26 ± 2.10	0.06
**MRCI score**			
Polypharmacy ⴕ	40/60 (66.7%)	12/18 (66.7%)	1.0
TOTAL score MRCI *	16.2 ± 9.4	15.0 ± 8.5	0.64

* Continuous variables (median, Interquartile Range, N); ⴕ Categorical variables (n/N, %); IC: Inform consent.

**Table 4:** Multivariate analysis.

Persistence of MMP		
Variable	OR (CI 95%)	*p* Value
Comorbidity that required a doctor follow-up	4.144 (1.188–14.453)	0.061
Heroin consumption	0.185 (0.053–0.652)	**0.028**
Job	2.884 (0.395–21.041)	0.381
**MRCI score ≥ 15**		
Homeless	2.037 (0.274–15.139)	0.487
Socio-family support	5.846 (0.337–101.262)	0.225
Comorbidity that required a doctor follow-up	7.200 (0.985–52.641)	**0.052**
HBV	7.921 (0.766–81.930)	0.083
Mental health disorder	15.928 (2.081–121.941)	**0.008**
Heroin consumption	0.231 (0.040–1.346)	0.10

OR: odds ratio; CI: confidence interval; HBV: hepatitis B virus.

## Data Availability

The datasets used and/or analyzed during the current study are available from the corresponding author on reasonable request.
